# A Multi-Species Phenotypic Screening Assay for Leishmaniasis Drug Discovery Shows That Active Compounds Display a High Degree of Species-Specificity

**DOI:** 10.3390/molecules25112551

**Published:** 2020-05-30

**Authors:** Laura M. Alcântara, Thalita C. S. Ferreira, Vanessa Fontana, Eric Chatelain, Carolina B. Moraes, Lucio H. Freitas-Junior

**Affiliations:** 1 Laboratório Nacional de Biociências (LNBio), Centro Nacional de Pesquisa em Energia e Materiais (CNPEM), Campinas, SP 13083-970, Brazil; lauramalcantara@outlook.com (L.M.A.); ferreira.tcs@outlook.com (T.C.S.F.); fontana_vanessa@yahoo.com.br (V.F.); 2 Departamento de Microbiologia, Instituto de Ciências Biomédicas, Universidade de São Paulo, São Paulo, SP 05508-900, Brazil; 3 Instituto Butantan, São Paulo, SP 05503-900, Brazil; 4 Drugs for Neglected Diseases Initiative, 1211 Geneva, Switzerland; echatelain@dndi.org

**Keywords:** *Leishmania*, leishmaniasis drug discovery, phenotypic screening, *Leishmania* species

## Abstract

High genetic and phenotypic variability between *Leishmania* species and strains within species make the development of broad-spectrum antileishmanial drugs challenging. Thus, screening panels consisting of several diverse *Leishmania* species can be useful in enabling compound prioritization based on their spectrum of activity. In this study, a robust and reproducible high content assay was developed, and 1280 small molecules were simultaneously screened against clinically relevant cutaneous and visceral species: *L. amazonensis*, *L. braziliensis*, and *L. donovani*. The assay is based on THP-1 macrophages infected with stationary phase promastigotes and posterior evaluation of both compound antileishmanial activity and host cell toxicity. The profile of compound activity was species-specific, and out of 51 active compounds, only 14 presented broad-spectrum activity against the three species, with activities ranging from 52% to 100%. Notably, the compounds CB1954, Clomipramine, Maprotiline, Protriptyline, and ML-9 presented pan-leishmanial activity, with efficacy greater than 70%. The results highlight the reduced number of compound classes with pan-leishmanial activity that might be available from diversity libraries, emphasizing the need to screen active compounds against a panel of species and strains. The assay reported here can be adapted to virtually any *Leishmania* species without the need for genetic modification of parasites, providing the basis for the discovery of broad spectrum anti-leishmanial agents.

## 1. Introduction

The leishmaniases are a group of vector-transmitted neglected tropical diseases caused by parasites of the genus *Leishmania.* They are endemic in more than 98 countries and territories, with an estimated incidence of 1.3 million new cases worldwide annually [1,2]. These diseases present a broad range of clinical manifestations and can vary from mild skin lesions (cutaneous leishmaniasis—CL) and mucous ulcers (mucocutaneous leishmaniasis—MCL) to systemic infection associated with visceral organ damage (visceral leishmaniasis—VL) [3,4]. Approximately 20 *Leishmania* species can be transmitted to humans by 78 species of the phlebotomine sand fly vector [5]. *Leishmania* species pathogenic to humans are classified into two subgenera: *Leishmania* (for example, *L. donovani* and *L. amazonensis*) and *Viannia* (*L. braziliensis*) [5,6]. *L. amazonensis* and *L. braziliensis* species primarily cause cutaneous leishmaniasis, while *L. donovani* causes primarily the visceral disease. *L. donovani* can also cause post-kala-azar dermal leishmaniasis (PKDL), a common complication of VL, characterized by macules, papules, and/or nodules in the skin [7].

The current pharmacological treatment for leishmaniasis is suboptimal, relying primarily on pentavalent antimonials, amphotericin B (in deoxycholate or liposomal formulations), and miltefosine [8,9]. Several issues have restricted the use of these drugs, such as toxicity resulting in severe side effects for patients [10,11,12], the relatively high costs, and difficult regimens, which often require parental administration and prolonged treatment; these barriers are particularly problematic given the socioeconomic status of affected populations [8,10,11,12,13,14,15,16,17,18,19,20]. Moreover, the treatment failure due to variability in drug efficacy depending on the *Leishmania* species and strain, clinical manifestations, and geographic regions [6,21], and in some cases, emergence of resistance to antileishmanial drugs [13,14,15,16,17,18] are other concerns that highlight the need for the development of new chemotherapy options for leishmaniasis that are efficacious and safe, have a short oral course treatment, and protect against resistance development. A limited number of chemical entities discovered for leishmaniasis have been advanced to pre-clinical and clinical phases, including aminopyrazole, pyrazolopyrimidine, oxaborole, and nitroimidazole compounds [22,23], and there is, therefore, a continual need for the discovery of new chemotypes, with distinct mechanisms of action.

This scenario has prompted pharmaceutical companies and research institutes to engage in screening millions of compounds by utilizing in vitro cell-based assays, which has resulted in: (i) the identification of potential chemical series with antileishmanial activity [24,25] and (ii) the discovery of new potential *Leishmania* targets for chemotherapy, such as proteasome [26], methionyl-tRNA synthetase [27], and cyclin-dependent kinase 12 [28]. However, most of the published work carried out by drug discovery programs and partnerships has focused on the viscerotropic *Leishmania donovani* species [24,29,30,31,32,33] with very few screening campaigns reported for dermotropic species [34,35].

As different species and strains within species often present phenotypic variability and require and/or allow for different culturing conditions, both in vitro and in vivo, several screening protocols have been reported using distinct host cells (both immortalized cell lines and primary cells), parasite stages for infection (promastigotes, axenic amastigotes, and ex vivo amastigotes), periods of drug incubation, and methods of detection and analysis [36]. These differences, while advantageous for tailoring experimental conditions to best address particular questions, greatly complicate the comparison of drug screening data obtained with different assays, as compound activity may be due, at least in part, to divergences in experimental conditions.

To address these issues, we have developed a standardized infection and drug screening assay for *Leishmania* species and aimed at further exploring the differences between species by comparing the results obtained from the screening of a diversity library against three clinically relevant *Leishmania* species: two cutaneous species, *L. amazonensis*, which causes the severe syndrome diffuse cutaneous leishmaniasis, and *L. braziliensis*, the species most often associated with the highly disfiguring mucocutaneous leishmaniasis, as well as the visceral species *L. donovani,* one of the species causing the often lethal visceral leishmaniasis.

## 2. Results

### 2.1. Development of a Multi-Species High Content Screening Assay

**Figure 1:**
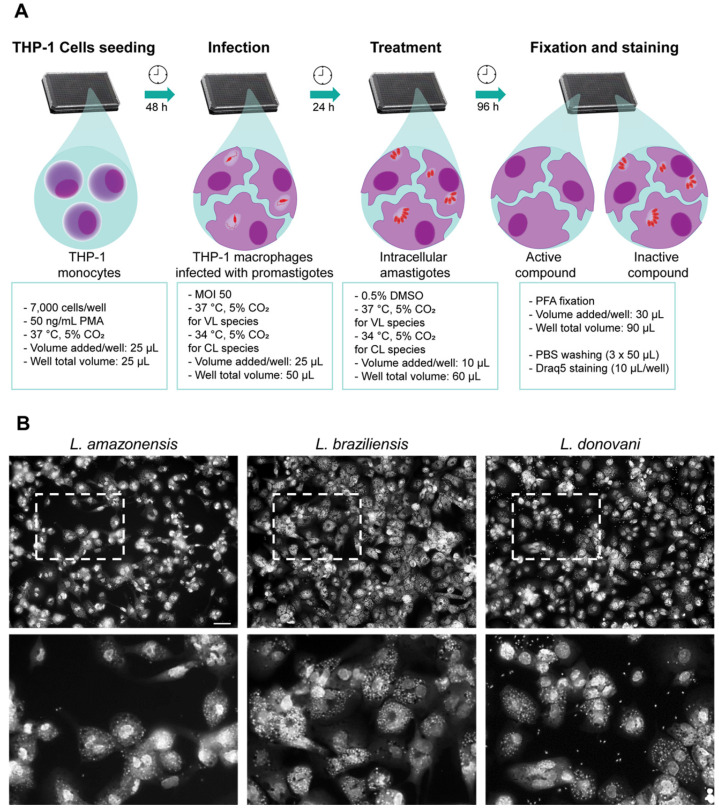
*Leishmania* high content assay. (**A**) Schematic representation of *Leishmania* multi-species high content assay. THP-1 cells were seeded and differentiated with phorbol 12-myristate 13-acetate (PMA) for 48 h, followed by infection with stationary-phase promastigotes. After another 24 h, compounds were added to infected cultures, and plates were incubated for 96 h. After this period, plates were fixed and stained. Images were acquired and processed in a High Content Screening instrument. VL, visceral leishmaniasis; CL, cutaneous leishmaniasis. (**B**) Representative images of *Leishmania*-infected THP-1 macrophages (multiplicity of infection (MOI) 50), 120 h post infection. Scale bar = 50 µm.

High content screening (HCS) has been largely used in the interrogation of small and large compound libraries for leishmaniasis drug discovery, as it is amenable to automation, enables compound testing against intracellular amastigotes, and results in the determination of antiparasitic activity and host cell selectivity within a single assay [29,30,31,37,38]. The semi-automated assay developed for this work is based on the infection of phorbol 12-myristate 13-acetate (PMA)-differentiated THP-1 macrophages with stationary-phase promastigotes (Figure 1A). Stimulation with PMA for 48 h was sufficient for differentiation of THP-1 into macrophage-like cells, as demonstrated by cell adherence, cytoplasmatic area enlargement and phagocytic capacity (data not shown). The maintenance of PMA in the culture medium upon infection and drug exposure ensured that most THP-1 cells would remain differentiated as macrophage-like cells (Figure S1). Because *Leishmania* species present different temperature tolerances for intracellular persistence and multiplication [39], infection with cutaneous species was performed at 34 °C as this increased infection substantially (Figure S2). A concentration of 0.5% DMSO was found to be well tolerated by THP-1 cells and all *Leishmania* species analyzed, as it did not affect the host cell number, the infection ratio, or the number of intracellular parasites (data not shown). At least 96 h of incubation with amphotericin B, herein used as a positive control, were necessary to eradicate or greatly reduce intracellular infection, and thus 96 h was set as the drug exposure window in the screening assay (Figure S3).

Given the phenotypic variability of *Leishmania*, there were morphological differences in cells infected with different species at the assay endpoint (Figure 1B), including cytoplasm area, size and staining of parasites, and distance between amastigotes. Thus, image analysis settings were optimized for each species to obtain appropriate cell segmentation and parasite detection. The general pipeline of image analysis in a sequential building block setup is demonstrated in Figure S4 and Table S1.

**Figure 2:**
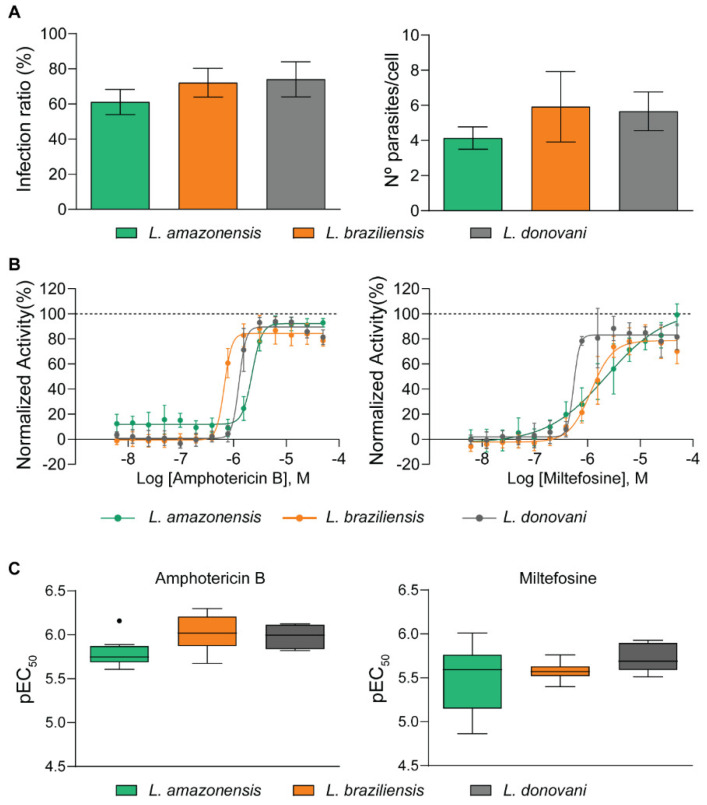
*Leishmania* high content assay parameters. (**A**) Quantitative parameters of *Leishmania* infection in terms of infection ratio (left) and the number of amastigotes/cells (right). (**B**) Concentration–effect curves of the reference compounds amphotericin B and miltefosine against *Leishmania* species. The *X*-axis indicates the log of compound concentration (molar), and the *Y*-axis indicates the normalized antiparasitic activity, which represents the inhibition of infection in relation to controls. EC_50_ values for amphotericin B were: 1.7 µM for *L. amazonensis*; 1.3 µM for *L. braziliensis*; and 0.7 µM for *L. donovani*. EC_50_ values for miltefosine were: 2.3 µM for *L. amazonensis*; 1.6 µM, for *L. braziliensis*; and 0.6 µM for *L. donovani*. As indicated in legend: *L. amazonensis* (green), *L. braziliensis* (orange), and *L. donovani* (grey). (**C**) Boxplots of ten independently determined pEC_50_ values of amphotericin B and miltefosine. The plot shows median (line within box), 25th and 75th percentiles (box), and minimum and maximum (whiskers). The black circle indicates an outlier.

The optimal conditions for assay and image analysis resulted in infection ratios higher than 60% and a detected number of parasites/infected cells higher than 4.5 for the three species (Figure 2A).

To validate the HCS protocol sensitivity, the dose–response curves of amphotericin B and miltefosine were evaluated against the three species. However, while amphotericin B was highly active against all species (reaching maximum efficacy of approx. 100% and EC_50_ values ranging from 0.7 to 2.0 µM), maximum efficacy for miltefosine varied from 78% to 100%, and potency varied up to 4-fold between species (Figure 2B). *L. donovani* was more sensitive to miltefosine (EC_50_ = 0.6 µM) when compared to *L. braziliensis* (EC_50_ = 1.6 µM) and *L. amazonensis* (EC_50_ = 2.3 µM).

To verify the reproducibility of the system and the consistency in activity of the reference drugs, data from ten independent experiments were assessed. Figure 2C shows a negligible variation in pEC_50_ values across experiments for all drug and species combinations, except for miltefosine in *L. amazonensis*, for which pEC_50_ values varied from 6.01 (EC_50_ = 0.97 µM) to 4.86 (EC_50_ = 14 µM).

### 2.2. Diversity Library Screening against Leishmania Species

One objective of this study was to investigate differences in the susceptibility of *Leishmania* species to a diversity-based library and how this might affect hit discovery. The commercial compounds library LOPAC, composed of 1280 pharmacologically active molecules, was screened against *Leishmania* species in two independent experiments. Screening of the three species was performed under the same experimental conditions (i.e., reagents, cell and parasite cultures/passages, detection/analysis method). Quantitative parameters of high content screening of *Leishmania* species are shown in Table 1. Similar Z’-factor values for the three species (0.65–0.77) and a high correlation between independent experiments (>0.8 for normalized activity and >0.7 for cell ratio) demonstrated the robustness and the reproducibility of the HCS assay (Figure S5).

**Figure 3:**
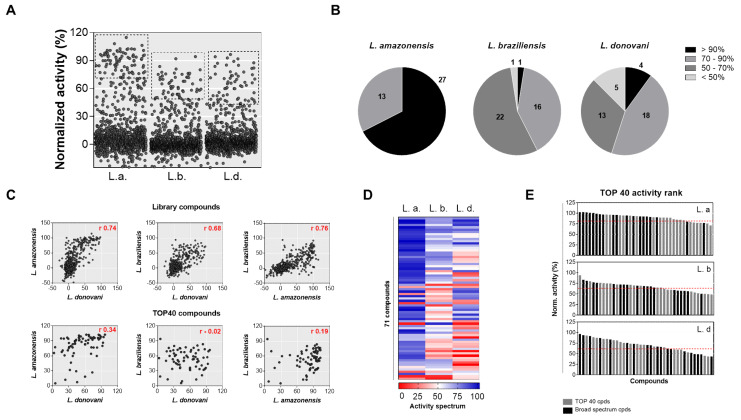
Primary screening of diversity-based library against *Leishmania* species panel. A library containing 1280 pharmacologically active molecules was screened at 50 µM against *L. donovani*, *L. braziliensis*, and *L. amazonensis* in two independent experiments. (**A**) Distribution of the whole library compounds per normalized activity. Data represent the mean normalized activity of each well from two independent experiments. The forty compounds that presented the highest activity and were nontoxic to human macrophages were selected as “top 40” and are contained in the dotted square. (**B**) Pie charts representing the distribution of the top 40 compounds per normalized activity for *Leishmania* species. The values inside the graphs represent the number of compounds. (**C**) Correlation of compound activity between the species in pairs, in terms of library compounds (top) and “top 40” compounds (bottom). The mean normalized activity from two independent experiments is plotted. Spearman rank correlation coefficients are shown in the right top corner of the graphs. (**D**) Heatmap of compound activity on *Leishmania* species infection. The color scale illustrates normalized activity in relation to controls: zero activity (red), 50% activity (blank), and 100% activity (blue). (**E**) Activity rank of top 40 compounds, for each *Leishmania* model: broad spectrum compounds (black) and top 40 compounds (grey).

The distribution pattern of compounds and controls per normalized activity was remarkably different between the species (Figure S6A), demonstrating variable sensitivity to the compound library. As result, the number of hits, defined as compounds with at least 50% mean normalized activity and 0.5 cell ratio, were: 61, 39, and 31 for *L. amazonensis*, *L. braziliensis*, and *L. donovani,* respectively (Figure S6B). The 40 most active compounds (top 40 compounds) with a cell ratio ≥ 0.5 (~3% of the library) were selected as hits for further experiments (Figure 3A).

*L. amazonensis* was the most sensitive species, with the top 40 compounds presenting a mean activity of 81.5% (ranging from 71% to 103%), followed by *L. donovani* and *L. braziliensis*, with a mean activity of 63.5% (43–97%) and 61.5% (49–94%), respectively. Moreover, analyzing the distribution pattern of the top 40 compounds (Figure 3B), approx. 70% of selected compounds presented activity > 90% in the *L. amazonensis* screen. On the other hand, compounds selected against *L. donovani* and *L. braziliensis* species were widely distributed into four activity classes (from < 50% to > 90%), and only four (10%) (*L. donovani*) and one (2.5%) (*L. braziliensis*) of the top 40 compounds had activity higher than 90%. Disregarding the duplicates, a set of 71 unique compounds was selected from the multi-species primary screenings.

To investigate differences in compound activity level between the species, correlations were generated for both the whole library and for the 71 active compounds (Figure 3C). High correlation was observed between species pairs for general library compounds: *L. braziliensis x L. amazonensis* (0.76), *L. donovani x L. amazonensis* (0.74), and *L. donovani x L. braziliensis* (0.68); however, no significant correlation was observed for the 71 active compounds (0.02 to 0.34).

From this set of 71 compounds, 41 (58%) were selected as a single species hit, 11 (15%) were selected as two species hits, and 19 (27%) were selected as hits in all three screens. The characterization of these compounds in terms of the activity spectrum is shown in Figure 3D. In *L. amazonensis*, most compounds presented activity > 75%, further demonstrating its higher sensitivity to the compound library. In contrast, *L. braziliensis* and *L. donovani* species were considerably less sensitive to compounds selected in other screens.

To verify if the 19 common hit compounds were also the most active compounds of each model, they were ranked by their activity (Figure 3E). While in the *L. amazonensis* model, eight shared compounds presented the highest activity, in the other two models, common compounds were broadly distributed in the activity ranking.

Taken together, these data highlight that there is a high species-specificity for *Leishmania* species, which impacted the selection of hit compounds from this primary screening.

### 2.3. Identification of Pan-Active Compounds

**Figure 4:**
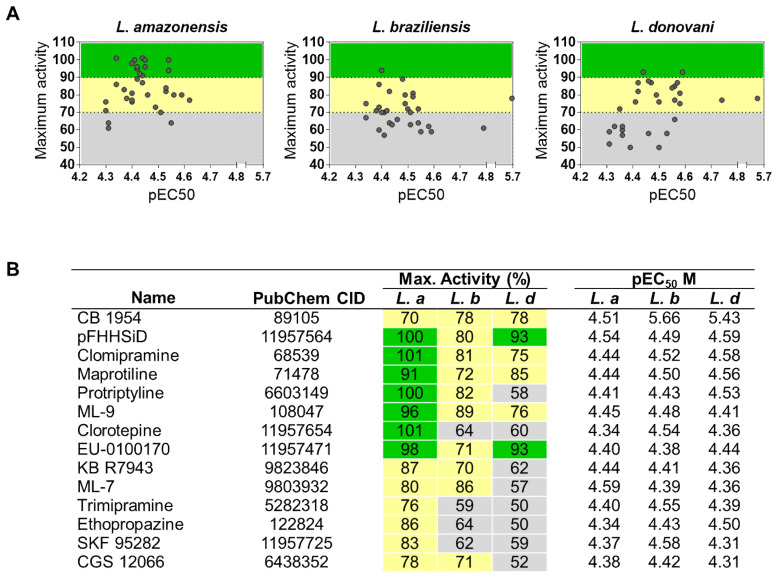
Confirmatory screening of a diversity-based library against *Leishmania* species panel. (**A**) Scatter plot distribution of compounds based on pEC_50_ values and maximum activity (%). (**B**) Activity profile of pan-leishmanial compounds in terms of potency and efficacy. Colors indicate: max. activity > 90% (green), 70% < max. activity < 90% (yellow), and max. activity < 70% (grey). Max. activity is the mean value of normalized activity percentage at 50 µM of four independent experiments. pEC_50_ = -log EC_50_ (M). pEC50 is the mean value of two independent experiments. *L.a* = *Leishmania amazonensis; L.b* = *Leishmania braziliensis*, and *L.d = Leishmania donovani.*

To validate and further characterize the activity of selected compounds from primary screening, two independent dose–response confirmatory screens were carried out. From the 40 compounds tested in each *Leishmania* assay, it was possible to determine EC_50_ values for 33 (82.5%), 31 (77.5%), and 29 (73.5%) compounds in *L. amazonensis*, *L. braziliensis*, and *L. donovani*, respectively, totaling a set of 51 compounds with confirmed activity (Figure 4 and Table S2). Overall, the mean EC_50_ values were 30 μM, i.e., pEC_50_ ~4.5 (Figure 4A). No compound with an EC_50_ value < 20 µM was found against *L. amazonensis*, whereas the most potent compounds, with species-specific activity, were carvedilol (EC_50_ = 16.2 µM) against *L. braziliensis* and indatraline (EC_50_ = 18.1 µM) against *L. donovani* (Table S2). CB1954 was the only compound that exhibited high potency against more than two species: *L. donovani and L. braziliensis* (EC_50_ of 3.7 and 1.6 µM, respectively), and moderate potency against *L. amazonensis* (EC_50_ of 31.0 µM)

In terms of efficacy, a list of 14 compounds with broad-spectrum activity was generated (Figure 4B). Two compounds (pFHHSi and EU–0100170) exhibited > 90% normalized activity against two species, and another 5 compounds (CB1954, Clomipramine, Maprotiline, Protriptyline and ML-9) presented pan-leishmanial activity (>70%). Compounds did not present cytotoxicity at the concentrations tested.

Thus, these results demonstrate variations in the potency and efficacy of compounds between *Leishmania* species, which resulted in a limited number of broad-spectrum hit compounds.

**Figure 5:**
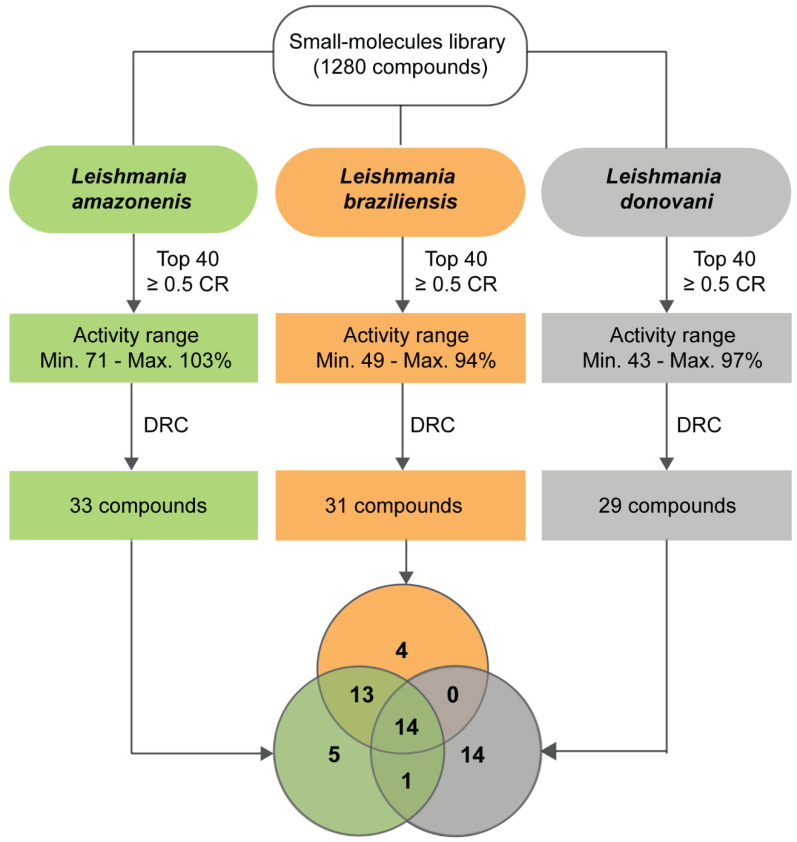
Summary of diversity-based library screening against *Leishmania* species. After the primary screening, the 40 most active compounds (top 40) with cell ratio (CR) ≥ 0.5 were selected in each model. The activity range of the selected compounds is shown in first squares. The top 40 compounds were then tested in dose–response experiments, and the number of compounds with determined EC_50_ is presented in second squares. The 51 compounds selected from all models are exhibited in the Venn Diagram, which shows their activity spectrum.

In summary, of the 51 compounds, 14 (27.5%) were broad-spectrum candidates (activity ≥ 50%). Another 14 (27.5%) compounds shared activity between two species and 23 (45%) were species-specific. *L. donovani* had the highest rate of specific compounds (48%), followed by *L. amazonensis* (15%) and *L. braziliensis* (13%). Of the set of active compounds active against *L. amazonensis* and *L. braziliensis* (37 compounds), 13 (35%) presented activity against both cutaneous species and were not shared with *L. donovani*. The library screening cascade is represented in Figure 5.

## 3. Discussion

High content assays have become the most relevant strategy for compound interrogation in leishmaniasis early drug discovery, driving the identification of potential antileishmanial chemotypes and the discovery of new molecular targets to be explored. HCS assays do not require a validated molecular target and simultaneously provide evidence of compounds’ activity against intracellular amastigotes—the parasite stage related to disease progression—and toxicity in human host cells. Most efforts in this area, however, have focused on the viscerotropic *L. donovani*, and it is not yet clear how much of the chemical matter discovered as antileishmanial hit compounds can be repurposed for cutaneous (or, alternatively, New World) species. In another words, it is important to determine whether current efforts focused on *L. donovani* are also sufficient for the discovery of antileishmanials for cutaneous leishmaniasis, or if drug discovery focusing on CL merited new library screening campaigns, that could eventually lead to the development of specific drugs for cutaneous species.

To investigate differences in the activity of chemically diverse compounds between *Leishmania* species that have been explored in a less systematic manner, we sought to compare the results of screening a diversity library against the species *L. amazonensis* and *L. braziliensis*, both etiological agents of cutaneous leishmaniasis, to those against *L. donovani.* To this end, we have standardized an HCS assay that virtually eliminates technical differences between species-specific assays, enabling robust comparison of data and compound activity between the different species. To our knowledge, this is also the first HCS described for *L. braziliensis.*

Similar values of Z’-factor and high intraspecies correlation between independent experiment runs indicate a high degree of robustness and reproducibility for the assays for all three species. We have successfully applied the same methodology to drug assays with *L. infantum* and *L. major*, demonstrating that this assay can be adapted to most, and perhaps all, *Leishmania* species that infect humans and are amenable to in vitro culture (data not shown). Different species also exhibited comparable values of infection ratio and number of parasites/infected cell, which strongly suggest that compound activity variability was unrelated to differences in infectivity of species but was rather related to intrinsic variation in drug susceptibility, which may also vary depending on the compound in question.

Comparisons showed that while amphotericin B was highly active against all species, miltefosine showed varied potency and efficacy, especially against *L. amazonensis*. Variations in miltefosine susceptibility in *Leishmania* have been associated with distinct plasma membrane composition in different species and lipid content, which seem to influence drug uptake and, consequently, their activity^6,38^. Similarly, Escobar et al. have shown that amastigote forms of *L. aethiopica* were 14-fold more sensitive to miltefosine than *L. major* [40]. A distinct pattern in miltefosine activity against New World and Old-World *Leishmania* species has also been reported, with a study showing that miltefosine was approximately 20 times more potent in vitro against *L. donovani* than against *L. amazonensis, L. braziliensis, L. guyanensis*, and *L. chagasi* [41].

These differences in susceptibility became more pronounced in the context of compound library screening. Our comparative screens demonstrate that, although strong correlation was observed in the general library activity comparing screen pairs of different species (correlation index values 0.68–0.76), no significant or weak correlation was observed in hit compound activities comparing different *Leishmania* species (correlation index values < 0.34). *L. amazonensis* was the most sensitive species in the primary screening and 6 hit compounds were able to reduce parasite infection to undetectable levels. Conversely, *L. braziliensis* and *L. donovani* screens presented a broadly distributed activity profile, and only a few hit compounds presented maximum efficacy. Even the 19 common hit compounds selected in all primary screens had a variable activity profile in the different species. Of these, several compounds presented a pattern of species-specificity, especially in the case of *L. donovani* (approx. 50% of hit compounds with confirmed activity were exclusive to *L. donovani*).

In this study, while 14 of the active compounds were shared between *L. amazonensis* and *L. braziliensis*, *L. donovani* shared one compound with *L. amazonensis* and no compounds with *L. braziliensis,* indicating an association with species that cause similar clinical manifestations. As *L. donovani* is frequently used in early drug discovery, it could have a crucial impact on the identification of either pan-leishmanial candidates or candidates targeting specifically cutaneous species. Altogether, these data demonstrate that assays with different species (and probably strains within species) should be included as early as possible in the screening cascade to determine the spectrum of compound activity. Moreover, these results support the idea that *L. donovani* might not be an adequate surrogate species to perform drug discovery for cutaneous leishmaniasis and that discovery programs aiming at CL could benefit from performing de novo screening with *L. amazonensis* and *L. braziliensis*.

Lamotte and collaborators [37] reported comparative phenotypic screening between *L. amazonensis* and *L. donovani*, in which ex vivo amastigotes were used to infect primary mouse macrophages. Results were similar to the data presented in the current study; out of 188 compounds of the “Leish-Box” library, five (~3%) showed anti-leishmanial activity at the micromolar range against both *Leishmania* species [27]. This suggests that the low correspondence between distinct species is not dependent on the parasite form used in the infection, the host cells, or even the library (considering chemically diverse libraries). However, the “Leish Box” is a set of compounds previously selected from *L. donovani*-infected THP-1 cells [24], and the fact that most compounds were inactive in this study once again demonstrates the complexity of comparing data from different protocols, methodologies, and laboratories.

Beyond differences between species, variation between strains belonging to the same species impacts susceptibility to drugs. Studies performed with 245 clinical isolates of different *Leishmnia Viannia* species showed an important relationship between genetic diversity, zymodeme, and geographic distribution, and susceptibility to miltefosine and pentavalent antimonials [42]. More recently, Hefnawy and colleagues compared laboratory-adapted strains and clinical isolates from a library screening campaign. From a set of 130 molecules selected against a laboratory-adapted strain (the “Leishbox” from GSK^24^), 45% were also active against two other clinical strains recently isolated from patients, including antimonial-resistant and -sensitive strains. Additionally, this study showed that the differential activity spectrum was dependent on compounds’ chemical series and structures [37].

Although most broad-spectrum compounds presented low to moderate potency against *Leishmania* (pEC_50_ ~4.5), their chemical structures represent scaffolds worth exploring for further optimization (Figure S7). These compounds may also be deployed as tools to identify molecular targets or mechanisms/pathways that are shared between different species, which may be further exploited for antileishmanial drug discovery. In another relevant approach, compounds with broad-spectrum activity could be applied as chemical probes to better understand conserved metabolic pathways involved in parasite–host cell interactions (e.g., establishment of infection, parasite survival, and persistence and multiplication inside the macrophages).

Of the hits that presented panleishmanial activity during this screening, only CB1954 demonstrated high potency against *L. braziliensis* and *L. donovani* (EC_50_ of 1.6 and 3.7 µM, respectively), and moderate potency against *L. amazonensis* (EC_50_ of 31.0 µM). Other compounds (Figure 4), despite their high efficacy, presented only marginal to moderate potency against *Leishmania*. CB 1954, a nitroheterocyclic prodrug, was previously reported as an antitrypanosomatidic agent [43,44,45], validating the results obtained with our assay. Nitroheterocyclic drugs are structurally characterized by one or more nitro substituents attached to an aromatic ring and have been assessed as potential antileishmanial candidates. For instance, fexinidazole, the oral treatment recently recommended by the European Medicines Agency (EMA) for sleeping sickness [46], was tested in a phase II clinical trial against visceral leishmaniasis; however, it failed to demonstrate efficacy in patients [47]. Delamanid, a nitro-dihydro-imidazooxazole derivative formerly reported as antimycobacterial agent, has been shown to be effective against *Leishmania* [48]. Additionally, another nitroimidazole, DNDi-0690, is in Phase I clinical trial [46].

In conclusion, the assay reported here is robust and can be applied to drug discovery for leishmaniasis with different species. Our data indicate that the species of choice for primary screening should be prioritized according to the intended leishmaniasis target and that screening against different species (and strains, when available) should be introduced early in the screening cascade to allow for selection of compounds with a broader spectrum of activity.

## 4. Methods

### 4.1. Reference Compounds and Library

Amphotericin B and miltefosine (Sigma-Aldrich) stock solutions were prepared by dissolving standardized powder in dimethyl sulfoxide (DMSO) at 20 mM. LOPAC^®^1280 library was purchased from Sigma-Aldrich (cat n. LO4200). The library was dissolved in DMSO at a concentration of 10 mM and was stored at −20 °C under low-humidity conditions.

### 4.2. Host Cell and Parasite Cultures

Host cell: The human acute leukemia monocyte cell line THP-1 was cultured in RPMI 1640 media, supplemented with 20% (*v*/*v*) heat-inactivated fetal bovine serum (FBS), 100 U/mL penicillin, and 100 μg/mL streptomycin. Cells were subcultured every 3–4 days to maintain density between 2 × 10^5^ and 1 × 10^6^ cells/mL, up to 10 passages, at 37 °C in a 5% CO_2_ humidified incubator. Cell stocks were obtained from the Rio de Janeiro Cell Bank—BCRJ (catalog. no: 0234).

Parasites: *L. donovani* (MHOM/IN/1980/DD8) promastigotes were kindly provided by Prof. Silvia Uliana, USP, Brazil, while *L. amazonensis* (MHOM/BR/1977/LTB0016) and *L. braziliensis* (MHOM/BR/1975/M2903) were obtained through the Fundação Oswaldo Cruz, Rio de Janeiro *Leishmania* repository—CLIOC. *Leishmania spp.* promastigotes were cultivated at 26 °C under rotation (30 rpm) in a shaking incubator, in media 199 with 40 mM Hepes, 0.1 mM adenine, 1 µg/mL biotin, 4 mM NaHCO_3_, 10% FBS (*v*/*v*) (20% for *L. braziliensis*), 100 U/mL penicillin, and 100 μg/mL streptomycin. Parasite cultures were subcultured every 3 days, for up to 6 passages. To prepare stationary-phase promastigotes, 1 × 10^6^ parasites/mL were placed into T75 flasks and maintained for 5–6 days without media replacement.

### 4.3. Intracellular Amastigotes Assay and Library Screening

THP-1 cells were plated onto 384-well assay plates (7000 cells/well, 25 μL) in RPMI complete media containing 50 ng/mL phorbol 12-myristate 13-acetate (PMA) and incubated at 37 °C/5% CO_2_ for 48 h. Cells were then infected with stationary-phase promastigotes at a multiplicity of infection (MOI) of 50 by adding 25 μL RPMI (without PMA reagent), containing 1.4 × 10^7^ parasites/mL. After 24 h of incubation at 37 °C (34 °C for cutaneous species), negative control-vehicle (0.5% DMSO), the positive control (10 μM amphotericin B), and compounds were added to the plate at a volume of 10 μL/well, bringing the total volume to 60 μL/well. For the primary screenings, compounds from the LOPAC library were tested at a concentration of 50 µM. For hit activity confirmation in dose response, compounds were ‘cherry-picked’ and serially diluted by a factor of two, starting at 50 μM. Assay plates were incubated at 37 °C or 34 °C/5% CO_2_. After 96 h of compound exposure, assay plates were fixed with paraformaldehyde (PFA) by adding 30 μL of 12% PFA in PBS, pH 7.4, followed by incubation for 15 min at room temperature. Plates were then washed three times with 50 μL of PBS, pH 7.4, and stained with 10 μL/well of 5 μM Draq5 in PBS, pH 7.4, for at least two hours before imaging.

### 4.4. High Content Image Acquisition and Analysis

Plates were imaged in Operetta High Content Imaging System (Perkin Elmer), version 3.1, at a 20× magnification and filters optimized for far-red fluorescence (fluorescence filter: 635 nM). A total of four images were acquired per well, which corresponded to 600–800 cells analyzed per condition. Quantitative readouts of the infection (total THP-1 cell number, total number of infected THP-1 cells, and number of parasites (spots) per infected cell) were determined by custom analysis building blocks in Columbus image analysis software (Perkin Elmer). Briefly, host nuclei and cells were segmented based on DNA staining, followed by the detection of individual parasites also based on DNA staining. Infected cells were determined as cells with one or more spots in the cytoplasmatic area. Details of the analysis workflow are described in the (Supplementary Information (Figure S4 and Table S1).

### 4.5. Data Analysis

HCS data were analyzed as previously described^29^. Briefly, the ratio of infected cells to the total number of cells was determined as the infection ratio (IR). The raw data for IR values was normalized to the negative (DMSO-treated infected cells) and positive (non-infected cells) controls to determine the normalized antiparasitic activity, expressed as a percentage in comparison to control wells. The cellular ratio was determined as the proportion between the host cell number in compound-treated wells and the mean cell number in infected control wells. Plates were submitted to quality control analysis using the Z’-factor^53^ and only plates with Z’-factor > 0.5 were considered for further analysis. Groups of different samples were analyzed using the ANOVA test followed by Tukey’s multiple comparison test. Studies of correlation were performed using Pearson or Spearman tests. Data from primary screenings were processed using Spotfire software, and the 40 most active compounds with cell ratio > 0.5 were selected for further confirmatory experiments. Dose–response data were processed with the Graphpad Prism software, version 6, using the sigmoidal dose–response (variable slope) nonlinear curve fitting function. EC_50_ values were determined by interpolation and defined as the compound concentration corresponding to 50% normalized activity.

## Figures and Tables

**Table 1:** Quantitative parameters of *Leishmania* multi-species high content screening.

Screening Parameters	*L. amazonensis*	*L. braziliensis*	*L. donovani*
Z’-factor	0.65 ± 0.04	0.76 ± 0.07	0.77 ± 0.05
Correlation index	0.86	0.88	0.89
CV of infected control (%)	11.74	11.4	13.5
EC_50_ Amphotericin B (µM)	2.10 ± 0.16	0.93 ± 0.45	0.82 ± 0.07

Values are mean ± standard deviation obtained from eight assay plates (4 plates/day). CV = coefficient of variation. Correlation index (Pearson test) was calculated based on the normalized activity of library compounds.

## Supplementary Materials

The following are available online, Figure S1: Establishment of the PMA differentiation protocol, Figure S2: Validation of assay temperature for infection with cutaneous *Leishmania* species, Figure S3: Determination of the drug exposure window, Figure S4: Representative images of high content analysis steps of *Leishmania* infection, Figure S5: Assay performance in diversity-based library screening for three *Leishmania* species, Figure S6: Comparison of library compound activity profiles between *Leishmania* species, Figure S7: Chemical structure of hit compounds that presented activity > 70% against all *Leishmania* species, Table S1: Details of analysis steps established in Columbus software, Table S2: Activity profile of top 40 compounds in dose-response confirmatory assays.
