# Supplementary material for: A Multi-Species Phenotypic Screening Assay for Leishmaniasis Drug Discovery Shows That Active Compounds Display a High Degree of Species-Specificity
Source: Molecules. 2020 May 30;25(11):2551. doi: 10.3390/molecules25112551 (PMC7321149; doi:10.3390/molecules25112551)
Supplement: Supplementary file 1 [file molecules-25-02551-s001.pdf]

A multi-species phenotypic screening assay for leishmaniasis drug discovery shows that active compounds display a high degree of species-specificity

Laura M. Alcântara [1,2,†], Thalita C. S. Ferreira [1,3,†], Vanessa Fontana [1,§], Eric Chatelain [4], Carolina B. Moraes [1,2,&,*] and Lucio H. Freitas-Junior [1,2,3,*]

[1] Laboratório Nacional de Biociências (LNBio), Centro Nacional de Pesquisa em Energia e Materiais (CNPEM), Campinas – SP, 13083-970, Brazil; lauramalcantara@outlook.com (L.M.A.); ferreira.tcs@outlook.com (T.C.S.F.); fontana_vanessa@yahoo.com.br (V.F.)

[2] Departamento de Microbiologia, Instituto de Ciências Biomédicas, Universidade de São Paulo, São Paulo – SP, 05508-900, Brazil

[3] Instituto Butantan, São Paulo – SP, 05503-900, Brazil;

[4] Drugs for Neglected Diseases *initiative*, Geneva, Switzerland; echatelain@dndi.org

Current address: [§]Department of Molecular and Clinical Pharmacology, University of Liverpool, Liverpool, L69 3GL, UK; [&]Department of Pharmaceutical Sciences, Federal University of São Paulo (UNIFESP), Diadema – SP, 09913-030, Brazil

* Correspondence: cbmoraes@unifesp.br (C.B.M.); luciofreitasjunior@gmail.com (L.H.F.-J.); Tel.: +55-11-4044-0500 – Ext. 3594

† These authors contributed equally to this work.

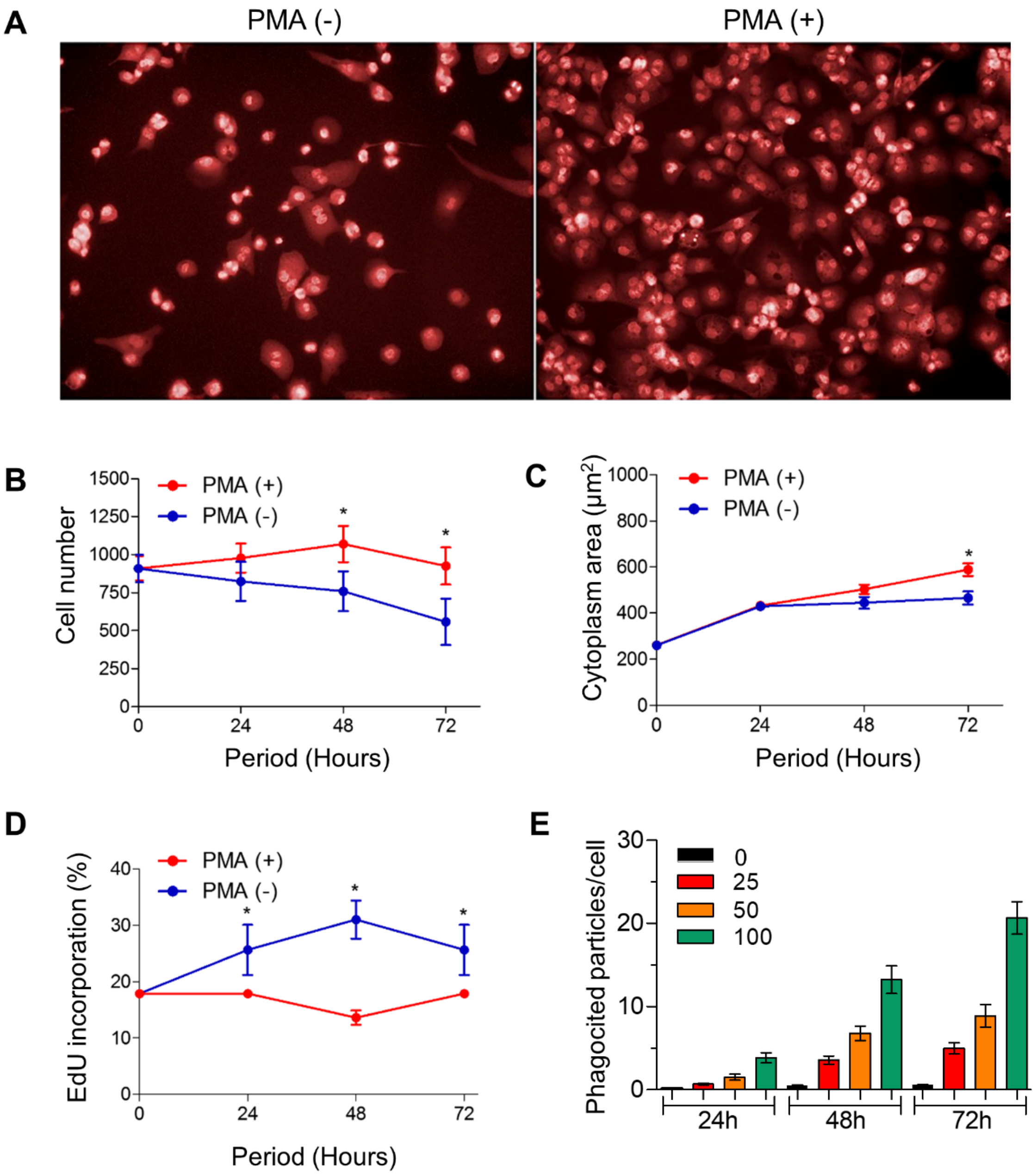

Figure S1. Establishment of the PMA differentiation protocol. After 48 h of differentiation with 50 ng/mL PMA, cells were maintained with [PMA (+)] or without [PMA(-)] reagent for a further 72 h. (A) Representative images of THP-1 macrophages cells at assay endpoint. Plate wells were stained with Draq5 dye and images were acquired with the Operetta High Content System, at a 20x magnification. Differences between PMA (+) (red lines) and PMA (-) (blue lines) cultures were also evaluated for: (B) host cell number, (C) mean cytoplasm area and (D) cellular multiplication ratio as determined by EdU incorporation. Asterisks (*) indicate that differences between PMA (+) and PMA (-) conditions were statistically significant ($p < 0.0001$). (E) Evaluation of THP-1 cells' phagocytic capacity after PMA differentiation protocol. Cells were activated with 50ng/ml PMA for 24, 48 or 72h, and then incubated with none (black), 25 (red), 50 (orange) or 100 (green) fluorescent zymosan particles per previously plated cell for 1h. The Y-axis shows the number of intracellular particles as determined by automated image analysis. Data shown are means and standard deviations of two independent experiments.

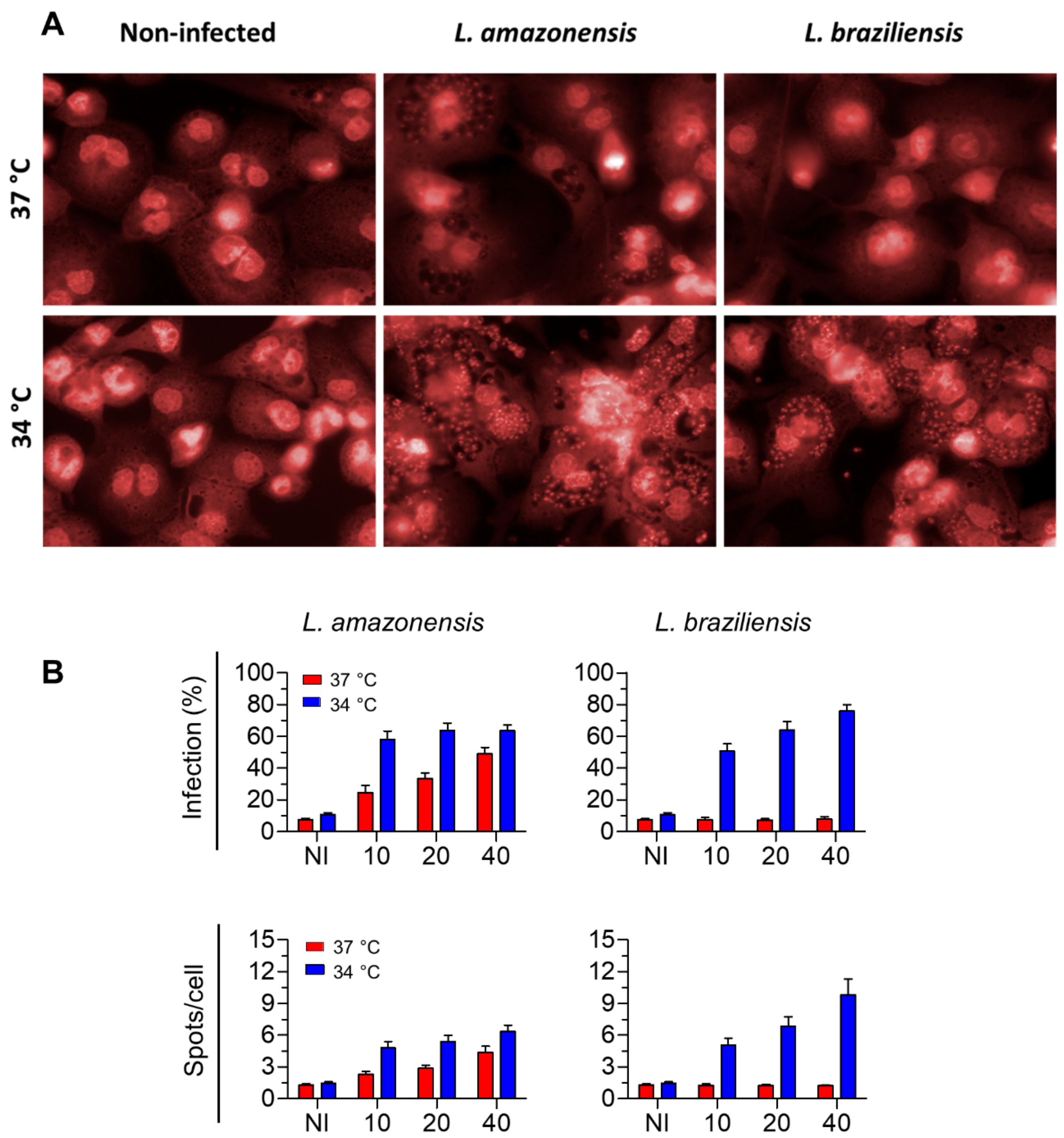

Figure S2. Validation of assay temperature for infection with cutaneous *Leishmania* species. (A) Representative images of THP-1 macrophages infected with *L. amazonensis* or *L. braziliensis* at 34 and 37 °C, after 96 h of infection. Infected cells were stained with Draq5 and images were acquired at 40x magnification. (B) Ratio of infected cells (Infection) and parasite number/infected cell (spots/cell), shown in the Y-axis, for different multiplicities of infection (MOIs) – 10, 20 and 40, shown in the X-axis – of *L. amazonensis* or *L. braziliensis* at 34 (blue) and 37 °C (red), 96 h post infection, as measured by automated image analysis. NI: non-infected. Data shown are means and standard deviations of three independent experiments.

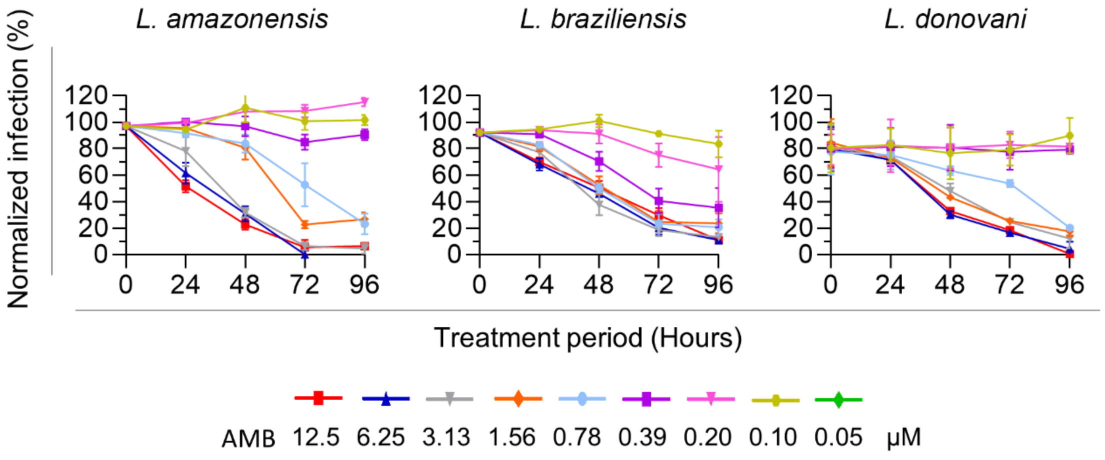

Figure S3. Determination of the drug exposure window. THP-1 cells were infected with *L. amazonensis*, *L. braziliensis* and *L. donovani* (MOI 50) for 24 h prior to addition of amphotericin B (previously serially diluted by a factor of 2, as indicated the by the colour legend). Activity was analyzed every 24 h, starting immediately after compound addition (time point 0 h), and up to 192 h. Data shown are means and standard deviations of two independent experiments.

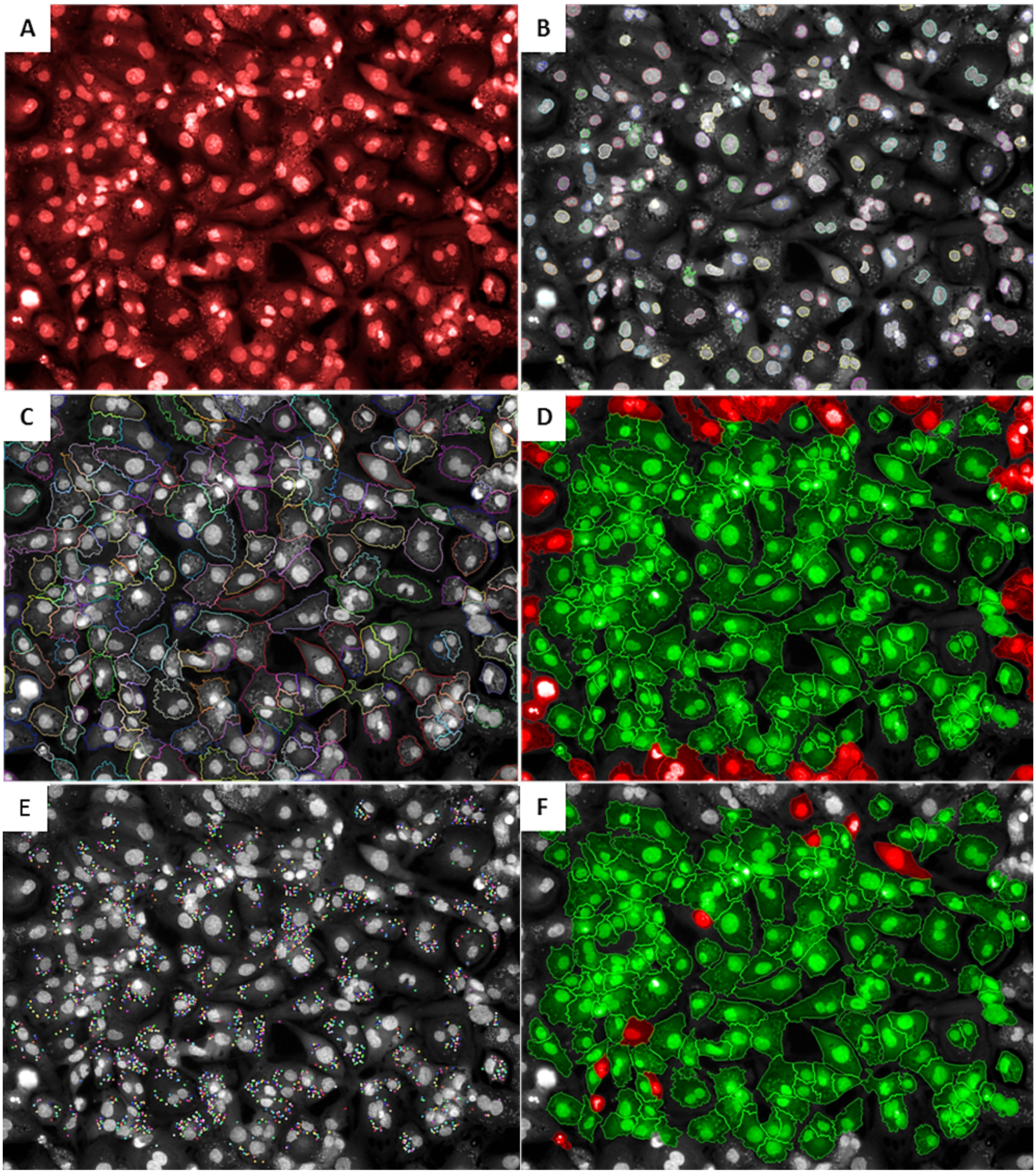

Figure S4. Representative images of high content analysis steps of *Leishmania* infection. Images of *Leishmania donovani*-infected THP-1 cells were used to determine the steps of building blocks in the Columbus analysis platform (Perkin Elmer). (A) raw images, (B) "find nuclei", software function used to determine the number of host cells (C) "find cytoplasm", used to segment individual cells, (D) "select population", applied to remove border objects, (E) "find spots", set to detect each intracellular amastigote and (F) "select population II", used to determine infected cells in the population.

Table S1. Details of analysis steps established in Columbus software.

Analysis steps	Objective	Parameters	Specifications
Find nuclei (method B)	Determines host cell nuclei and count cellular population.	Common Threshold	Determines the lowest level of pixel intensity for the whole image that belong to the nuclei
		Area	Tuning the merging and splitting of nuclei
		Split factor	Defines if large objects will be split into two or more smaller objects
		Individual Threshold	Determines the intensity threshold for each object individually
		Contrast	Sets the lowest contrast threshold for detected nuclei
Find cytoplasm (method B)	Delimits the host cell cytoplasm and segments the cells as individuals	Common threshold	Defines the borders of the cytoplasm
		Individual threshold	Determines the intensity threshold for each object individually
Select population I	Excludes cells not completely inserted in the image field	Common filters	Removes the border objects
Find spots (method C)	Detects and counts intracellular spots (parasites)	Radius	Sets an upper threshold for spot radius
		Contrast	Sets the lowest contrast threshold
		Uncorrected spot to region intensity	Defines the lowest threshold for spot to region intensity
		Distance	Determines the minimal distance between two adjacent spots
		Spot peak radius	Determines the radius of the disk over which the spot peak intensity is integrated
Select population II		Filter by property	Detects infected cells within the total population
Define results	Provides multiple parameters: number of host cells, infection ratio, number of parasites/infected cells		Sets which information will be provided from the image analysis.

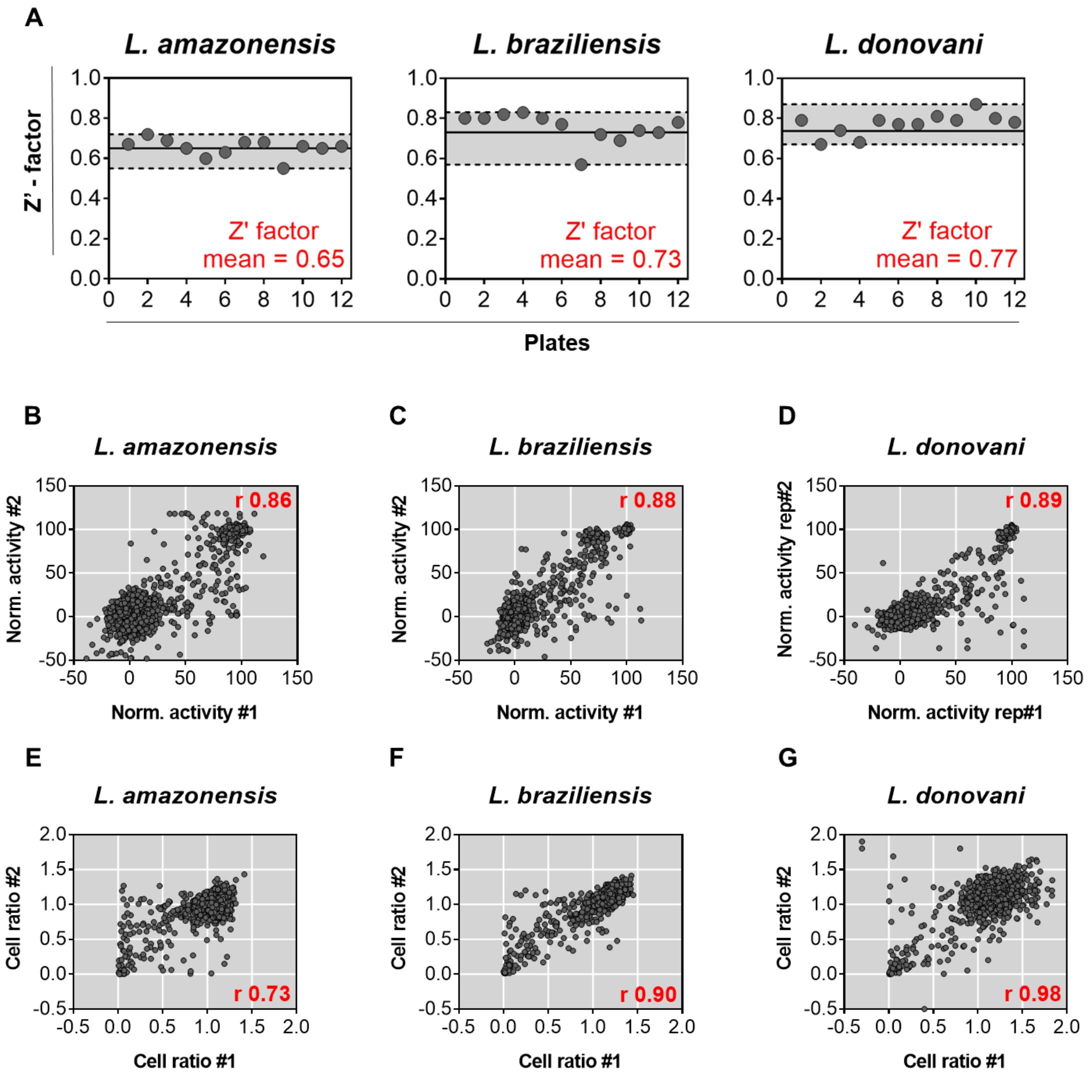

S5. Assay performance in diversity-based library screening for three *Leishmania* species. (A) Z'-factor values for assay plates, considering run 1 (plates from 1 to 6) and run 2 (plates from 7 to 12). For each run, four library compounds plates + two dose-response plates were prepared. Black lines indicate mean Z'-factor value and dotted black lines indicate both the maximum and the minimum values. (B), (C) and (D) Correlation of independent experiment runs for *L. amazonensis*, *L. braziliensis* and *L. donovani* screens, in terms of compounds' normalized activity. (E), (F) and (G) Correlation of independent experiment runs for *L. amazonensis*, *L. braziliensis* and *L. donovani* screens in terms of cell ratio. Dots represent each single tested well and Pearson correlation coefficients are shown in the right top/bottom corner of graphs (number is given in red). Experiment runs: run 1 (#1) and run 2 (#2).

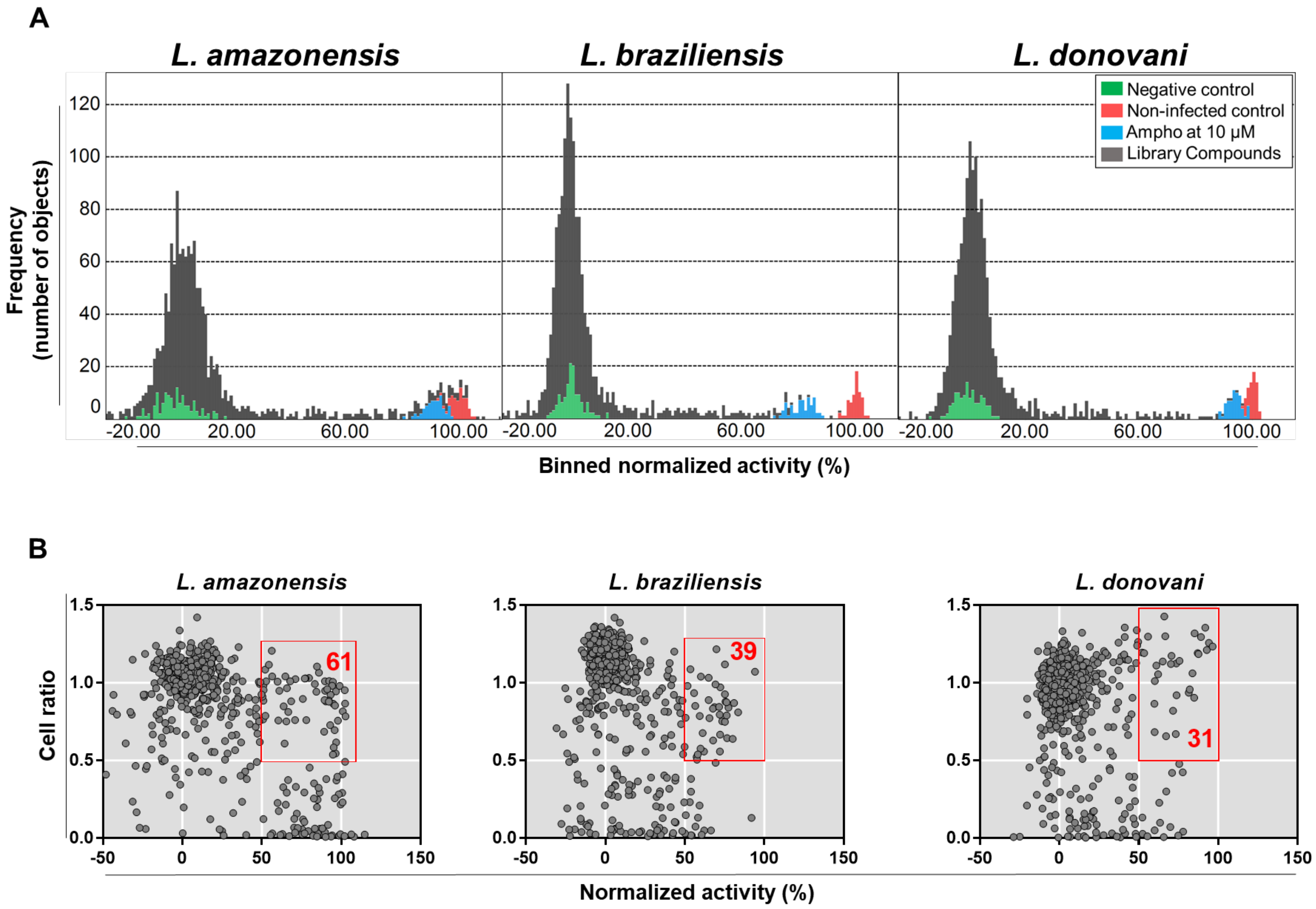

Figure S6. Comparison of library compound activity profiles between *Leishmania* species. (A) Controls and library compound distribution per normalized activity. X- axis indicates the binned normalized activity (%) and Y-axis shows the number of compounds per bin. As shown in the legend: negative control – infected and vehicle-treated wells (green), non-infected control (red), positive control – 10 µM amphotericin B (blue) and compounds (grey). Graphs represent data from two independent experiments. (B) Whole library compounds in terms of normalized activity (X-axis) and cell ratio (Y-axis). Dots represent mean values of each single tested well. The red squares highlight the compounds with activity > 50% and cell ratio > 0.5.

Table S2. Activity profile of top 40 compounds in dose-response confirmatory assays.

#	Name	PubChem CID	Max. Activity (%)			pEC_{50} M		
			L. a	*L. b*	*L. d*	*L. a*	*L. b*	*L. d*
1	**CB 1954**	**89105**	70	78	78	4.51	5.66	5.43
2	**pFHHSiD**	**11957564**	100	80	93	4.54	4.49	4.59
3	**Clomipramine**	**68539**	101	81	75	4.44	4.52	4.58
4	**Maprotiline**	**71478**	91	72	85	4.44	4.50	4.56
5	**Protriptyline**	**6603149**	100	82	58	4.41	4.43	4.53
6	**ML-9**	**108047**	96	89	76	4.45	4.48	4.41
7	**Clorotepine**	**11957654**	101	64	60	4.34	4.54	4.36
8	**EU-0100170**	**11957471**	98	71	93	4.40	4.38	4.44
9	**KB R7943**	**9823846**	87	70	62	4.44	4.41	4.36
10	**ML-7**	**9803932**	80	86	57	4.59	4.39	4.36
11	**Trimipramine**	**5282318**	76	59	50	4.40	4.55	4.39
12	**Ethopropazine**	**122824**	86	64	50	4.34	4.43	4.50
13	**SKF 95282**	**11957725**	83	62	59	4.37	4.58	4.31
14	**CGS 12066**	**6438352**	78	71	52	4.38	4.42	4.31
15	**Carvedilol**	**2585**	100	61		4.45	4.79	
16	**Desipramine**	**65327**	80	75		4.56	4.49	
17	**Benztropine**	**238053**	73	72		4.49	4.54	
18	**Vinblastine**	**11957719**	64	66		4.55	4.46	
19	**Propionylpromazine**	**24351**	80	70		4.46	4.51	
20	**Nortriptyline**	**441358**	82	63		4.53	4.44	
21	**Iminoaspartate**	**2820**	95	79		4.42	4.52	
22	**Amoxapine**	**2170**	89	63		4.42	4.51	
23	**Cyproheptadine**	**13770**	84	73		4.53	4.39	
24	**Fluphenazine**	**67356**	96		82	4.42		4.42
25	**Bromocryptine**	**31100**	92	57		4.43	4.41	
26	**Promethazine**	**6014**	81	70		4.40	4.40	
27	**NS8593**	**71311765**	77	75		4.40	4.34	
28	**1H-Imidazole**	**2818**	61	60		4.31	4.39	
29	**Indatraline**	**10314472**			77			4.74
30	**5HPP-33**	**11723708**	77			4.62		
31	**CyPPA**	**909822**		59			4.59	
32	**Thioridazine**	**66062**			81			4.58
33	**Loperamide**	**71420**			87			4.57
34	**Perifosine**	**148177**			66			4.56
35	**Perphenazine**	**4748**			77			4.56
36	**Triflupromazine**	**66069**			84			4.55
37	**Cerestat**	**60839**	94			4.54		
38	**Paroxetine**	**62878**			76			4.50
39	**Metergoline**	**28693**			80			4.49
40	**Methiothepin**	**3039995**			87			4.47
41	**L-741626**	**133633**			58			4.46
42	**Flupenthixol**	**5282483**			88			4.46
43	**Trifluoperazine**	**66064**			87			4.42
44	**EU-0100695**	**11957588**		57			4.41	
45	**Dalasetron**	**6918119**		94			4.40	
46	**CID2858522**	**2858523**			72			4.35
47	**Promazine**	**5887**		67			4.34	
48	**NSC 10120**	**672296**			62			4.33
49	**EU-0100484**	**11973707**	64			4.31		
50	**LP 44**	**11225543**	76			4.30		
51	**SKF 525A**	**65341**	71			4.30		

Max. Activity is the mean percentage value of normalized activity at 50 µM of four independent experiments. pEC50 is the mean value of two independent experiments. Colour code: max. activity > 90% (green), 70% < max. activity < 90% (yellow) and max. activity < 70% (grey). Blank spaces represent activity < 50%. $pEC_{50} = -\log EC_{50}$ (M).

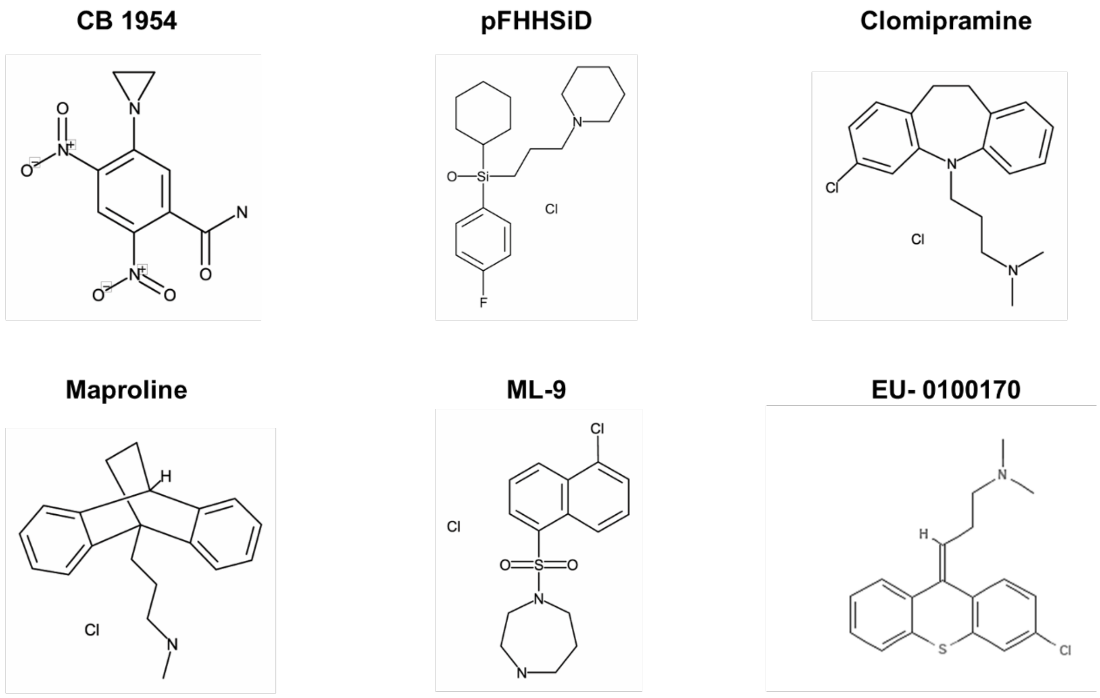

Figure S.7. Chemical structure of hit compounds that presented activity > 70% against all *Leishmania* species.
